# Cut, Root, and Grow: Simplifying Cassava Propagation to Scale

**DOI:** 10.3390/plants13040471

**Published:** 2024-02-06

**Authors:** Samar Sheat, Edda Mushi, Francisca Gwandu, Mouritala Sikirou, Patrick Baleke, Siraj Ismail Kayondo, Heneriko Kulembeka, Najimu Adetoro, Stephan Winter

**Affiliations:** 1 Plant Virus Department, Leibniz Institute DSMZ-German Collection of Microorganisms and Cell Cultures, 38124 Braunschweig, Germany; stephan.winter@dsmz.de; 2 International Institute of Tropical Agriculture (IITA), Dar es Salaam P.O. Box 3444, Tanzania; e.mushi@cgiar.org (E.M.); s.kayondo@cgiar.org (S.I.K.); 3 Tanzania Agricultural Research Institute, TARI Ukiriguru, Mwanza P.O. Box 1433, Tanzania; francisca.gwandu@gmail.com (F.G.); kulembeka@yahoo.com (H.K.); 4 International Institute of Tropical Agriculture (IITA), Kalambo Bukavu P.O. Box 4163, Democratic Republic of the Congo; m.sikirou@cgiar.org (M.S.); n.adetoro@cgiar.org (N.A.); 5 Association Volontaire pour le Développement (AVPD), Karenzu, Luvungi, Itara 1, Democratic Republic of the Congo; p.baleke@gmail.com

**Keywords:** leaf bud, sprouting, rooting, multiplication, cost-effective, disease-free, water

## Abstract

Cassava (*Manihot esculenta* Crantz) is an essential crop with increasing importance for food supply and as raw material for industrial processing. The crop is vegetatively propagated through stem cuttings taken at the end of the growing cycle and its low multiplication rate and the high cost of stem transportation are detrimental to the increasing demand for high-quality cassava planting materials. Rapid multiplication of vegetative propagules of crops comprises tissue culture (TC) and semi-autotroph hydroponics (SAH) that provide cost-effective propagation of plant materials; however, they contrast the need for specific infrastructure, special media and substrates, and trained personnel. Traditional methods such as TC and SAH have shown promise in efficient plant material propagation. Nonetheless, these techniques necessitate specific infrastructure, specialized media and substrates, as well as trained personnel. Moreover, losses during the intermediate nursery and adaptation stages limit the overall effectiveness of these methods. Building upon an earlier report from Embrapa Brazil, which utilized mature buds from cassava for rapid propagation, we present a modified protocol that simplifies the process for wider adoption. Our method involves excising single nodes with attached leaves from immature (green) cassava stems at 2 months after planting (MAP). These nodes are then germinated in pure water, eliminating the need for specific growth substrates and additional treatments. After the initial phase, the rooted sprouts are transferred into soil within 1–8 weeks. The protocol demonstrates a high turnover rate at minimal costs. Due to its simplicity, cost-effectiveness, and robustness, this method holds significant promise as an efficient means of producing cassava planting materials to meet diverse agricultural needs.

## 1. Introduction

Cassava plays a vital role in ensuring food security in tropical and subtropical regions and its importance is increasing because of the growing demand for cassava raw materials (starch) for industrial processing [1]. The crop is resilient to adverse environments [2], relatively undemanding, but responding well to increased agricultural inputs [3]. 

Cassava stands as a central focus for agricultural intensification in Sub-Saharan Africa, with the primary objective of augmenting both food security and income. Its significance lies in its dual-use attributes, serving as a crucial component of the food supply and holding substantial potential as a bioenergy crop [4]. Notably, climate suitability projections [5] suggest that, in contrast to other staple crops facing significant negative impacts from climate change, cassava exhibits remarkable resilience. Its increasing climatic suitability across most Africa regions positions cassava as a robust and sustainable solution for ensuring a reliable food supply [5]. All research and development efforts are geared towards improving cassava’s resilience against abiotic and biotic stresses and to exploit the crop’s productivity. To address the challenges in cassava breeding, the Next Generation Cassava breeding project (NextGen Cassava) (www.nextgencassava.org, accessed on 10 January 2024) was inaugurated in 2012, bringing together researchers from around the world into a consortium to accelerate cassava genetic improvement, develop new breeding tools, exchange materials and approaches to modernize and speed up cassava breeding, and to develop better varieties that meet the demands of diverse markets. The ultimate aim of all endeavors to improve the crop was/is the delivery of improved varieties and to ensure the widespread availability of high-quality farmer-preferred cassava materials with increased yield and resistance to diseases, making them accessible to the community at scale and reasonable costs.

Cassava is propagated through stem cuttings taken at the end of each growing cycle to start a new crop [6]. The low multiplication rate inherent in this traditional propagation method impedes meeting the increasing demand for high-quality cassava planting materials of improved disease-free and vigorously growing varieties. Thus, an efficient plant-multiplication system that produces cassava planting materials to scale at high rates and short cycles is needed to provide farmers with improved materials at reasonably low costs to replace inferior varieties. Systems for the rapid multiplication of vegetative propagules of crops comprise laboratory methods TC on sterile media [7,8,9] and SAH [10] (https://www.SAHTecno.com, accessed on 10 January 2024). The methods are well-established for bananas, cassava, yams, and other important crops. High multiplication rates and cost-effective propagation of the plant materials, however, contrast the need for specialized infrastructure, specific materials (substrates), and trained personnel to sustain the production of the plant materials in both labs and fields. Furthermore, the nursery materials have to reach the farm and their considerable losses at the intermediate nursery and adaptation step limit the otherwise high efficiency of the methods.

In our NextGen Cassava project, “Advancing Cassava Virus Resistance”, we implemented an extensive screening process to assess virus resistance in cassava germplasm and hybrid cassava lines. To achieve this, we employed a highly efficient infection and screening workflow [10] that was specifically designed for the purpose. The demand for testing thousands of cassava genotypes necessitated the development of a robust multiplication system capable of generating numerous clones from individual lines, serving as biological replicas. Initially, our strategy involved the rapid multiplication of single nodes excised from plantlets. These nodes were subjected to surface sterilization and cultured on growth media in a tissue culture environment, allowing for the production of sufficient plantlets within a short timeframe. However, despite its initial success, this approach proved a rate-limiting step. The need for surface sterilization of plant material before culturing on sterile media, coupled with the subsequent transplantation of TC into soil, hardening, and subsequent weeks of growth before testing, was time-consuming and inefficient. Recognizing this bottleneck, we explored alternative propagation methods to identify more straightforward and expeditious techniques to swiftly generate a large number of cassava stems for screening.

A method to produce cassava plantlets from mature nodes excised, with leaf attached, from cassava stems to initiate sprouting and root development was reported by Neves et al., 2020 [11]. In this method, the leaf buds were treated with agrochemicals prior to transplanting in a specific growing medium adjusted with nutrients [11]. The method we present here is built upon this earlier method; however, it omits any pre-treatment or the use of specific media. In our method, single nodes with leaves attached are excised from immature (green) cassava stems placed in plastic drinking cups with water added to initiate sprouting. After 1–8 weeks, rooted sprouts are transferred to soil for further growth.

Our emphasis was to present a simple and easy-to-follow protocol to produce cassava plantlets that can be readily adopted by nurseries for cassava propagation on commercial scale. The method does not require complex growing media, has low maintenance requirements, and the entire process does not incur costs. We refrained from using any media, growth-promoting, or anti-microbial chemicals and utilized pure tap water instead. A recycled plastic bottle filled with water to contain the cuttings for sprouting and no specific setup contrasted the earlier method report that used a complex mix of vermiculite and washed sand in a 1:1 ratio in the upper segment of the plastic tube (1/4 of the total volume) and, in the lower part of the plastic tube, vermiculite, soil, and coconut fiber in a 1:2:1 ratio, supplemented with 15 mg of single superphosphate and 15 mg of ammonium sulfate (comprising 3/4 of the total volume). Furthermore, we initially tested our simple set-up under controlled conditions but, subsequently, tried its application in field situations. This differs from the earlier approach, which relied on controlled humidity (70 ± 5%) and temperature (28 ± 2 °C). We show that even using immature green stems as sources for buds ensures high turnover, the simple method bares minimal costs and there are almost no losses in this robust process to produce plantlets. Furthermore, employing plants as mother plants just 2 MAP has significantly expedited the propagation process, contrasting the previous method that relied on plants at the 4 and 6 MAP stages.

The broad applicability of our method was evaluated by propagating 160 cassava genotypes to produce cassava clones for our virus screening. It was not our intention to compare growth parameters, e.g., plantlet height or dry mass; however, we focused our evaluation solely on a broader application of the rapid propagation method to produce cassava plantlets from a diversity of genotypes.

Because of its feasibility and robustness, we consider that this technique (Kitovu Jani—center leaf) warrants a broader application as an efficient method for producing cassava planting materials on a large scale.

## 2. Results

Our NextGen Cassava virus research framework needed an efficient method to rapidly produce clones from individual cassava seedlings to screen each unique genotype for resistance against cassava mosaic viruses and cassava brown streak viruses as well as reducing cassava breeding cycle length. The successful method, Kitovu Jani, was subsequently introduced to our research institutes in Africa to assess the feasibility of the technique for providing cassava planting materials on a large scale, on site, and for direct access and distribution to farmers.

### 2.1. Propagation of Cassava Using Single Nodes

**Figure 1:**
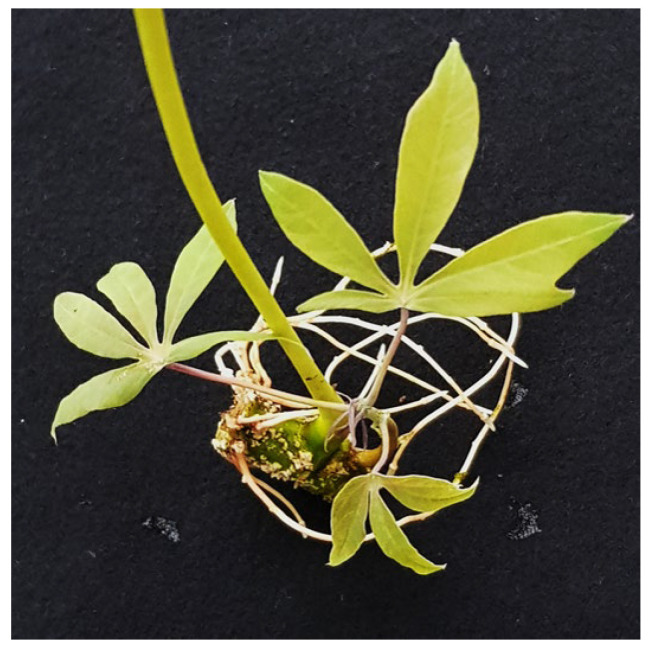
A cassava plantlet generated from a single bud approximately three weeks after the nodal cut was taken. Note that the petiole from the old leaf is still attached to the node.

Nodal cuttings from 149 seedlings (Table S1) placed in tap water for germination started to sprout after around 5 to 8 days. Root development started much later and, depending on the genotype, could take up to 2 weeks until small roots became visible. Differences among cassava genotypes were found; however, all buds developed into new sprouts within 2 weeks. From each of the 149 seedlings, 5 to 8 single-node cuttings were taken for germination; none failed to germinate and all developed leaves and roots. Cassava plantlets (Figure 1) transplanted into the soil grew vigorously and no losses were encountered during this soil adaptation process.

### 2.2. Propagation Efficiency under African Conditions

**Figure 2:**
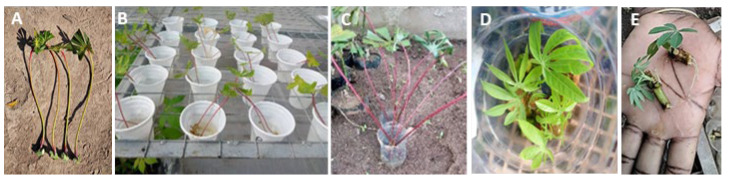
Cassava propagation using Kitovu Jani method. Single nodes with a leaf attached excised from green stems (**A**), germination in drinking cups (**B**) in IITA Tanzania, plastic bottle in TARI Tanzania (**C**), and IITA Kalmbo (**D**). Sprouts and roots germinated from a single bud node at AVPD DR Congo (**E**).

At IITA Tanzania Kwembe, 5 improved varieties, TARICASS4, Pwani, Mkuranga1, Kiroba, and Kizimbani, were subjected to Kitovu Jani with 100 petiole buds taken from each variety and placed into plastic drinking cups with water added that was changed every 2 days (Figure 2A,B). Pwani produced roots and shoots in 5 days, followed by TARICASS4 and Mkuranga1. The superior performance of Pwani, TARICASS4, and Mkuranga1 resulted in a survival rate of 80% and 98%, respectively. In contrast, Kiroba and Kizimbani had a delayed shoot and root formation, resulting in survival rates of less than 50%. Nevertheless, all plantlets having roots and shoots once transplanted into the soil in black polythene tubes survived and developed well. Differences in root and shoot formation were also recorded for the DSC breeding lines. A high survival rate of 86–95% was observed for DSC 673 and DSC 525 while DSC 510 and DSC 493 had only 60% and 50% survival rates, respectively.

At TARI, around 50 plantlets from each breeding line, KBH2016B/521, DSC 493, DSC 525, and KBH2016B/504, were subjected to Kitovu Jani (Figure 2C). Sprouting and leaf development much preceded the development of roots and, because of the delay in root formation, the plantlets were already transplanted into the soil when roots became visible after 5 days. Initially, the nodal cuttings were left in water for 7 to 14 days to force more roots. However, this resulted in a high death rate, probably due to contamination and high temperatures in the greenhouse. Despite this early moment of planting plantlets after 5 days germination in water, around 88% of DSC 525 and 100% for KBH2016B/521 developed into plantlets, while only 50% of KBH2016B/504 and DSC 493 were transplanted into soil to develop and grow into plants.

At IITA DR Congo, 3 cassava varieties, Ilona, Lumonu, and Mundola, were used in this experiment to grow 6 to 8 single-node cuttings for each of the varieties (Figure 2D). The water was replenished to prevent algal growth and, finally, transplanted to local peat with a survival rate around 75%. At AVPDA, around 25 plantlets from DSC 510, DSC 525, and DSC 673 were grown from around 40 single-node cuttings (Figure 2E). However, losses up to 30% were recorded and further optimization is needed to avoid water contamination and find a planting medium better suited for sensitive plantlets to grow.

In all cases, the low germination/survival was mostly attributed to water quality, excess water, contamination of water, and excess heat. This aspect of the method has to be given most attention and, while it is very simple to cut and grow, all phases of the method cannot be left unattended.

## 3. Discussion

The high-throughput screening protocol we have developed at the DSMZ Plant Virus Department to identify virus resistance in cassava crosses between cassava parents having contrasting resistances, either against cassava mosaic viruses or against cassava brown streak viruses [12], required multiple clones from seedlings to be subjected to virus infection bio-assays. Our conventional methods for propagation of cassava, comprising TC and subsequent transfer of the plantlets into soil, proved to be the rate-limiting factor of our workflow because nodal cuttings of the seedlings developing from the individual seeds were to be propagated first under sterile conditions in TC and, after that, transferred to soil and hardened. We tried several alternative propagation methods, including rooting of single-node cuttings in a plastic tunnel and SAH, a rapid propagation method for cassava developed and promoted by IITA (https://propas.iita.org/en/solutions/semi-autotrophic-hydroponics-for-cassave-multiplication/58/details/, acceded on 10 January 2024). These propagation methods resulted in an increased number of cassava clones; however, the growth rate to reach the bio-assay stage and the considerable losses of SAH during soil transfer proved suboptimal for the purpose.

The bud leaf technique Kitovu Jani we describe here presents a modification and radical simplification of the leaf bud method reported by [11] in which a substrate to foster shoot and root development of nodal cuttings and treatment with fungi/insecticides before planting was used. The Neves [11] report presented a useful guide to the best choice of nodal cuttings from cassava plants at different growing stages from 4 MAP to 12 MAP; however, we show that cassava propagation can be effective already at 2 MAP and starting with immature buds to increase the turnover. And, indeed, when nodal cuttings of cassava were submerged in pure water only, without substrate growing media supplemented with nutrients and antimicrobials as outlined in the earlier report, the rapid sprouting and shoot and root development (Figure 1) proved the effectiveness of this simple propagation method. While a slight increase in growth with fertilizer and antimicrobials was reported by Neves [11] it is crucial to recognize that this enhancement depends on the specific genotype. Using a rooting soil to grow the plantlets under glasshouse conditions resulted in 100% success with no losses when around 1500 single-node cuttings from 149 individual seedlings were rooted, which expands the spectrum of genotypes. This prompted us to transfer the method to the fields and to our partners to evaluate its feasibility for a broader application to provide high volumes of cassava materials of high quality, being disease-free, and with high vigor.

Testing over 160 cassava genotypes under various environmental and growth conditions underscores the broader applicability of the method.

An efficient cassava multiplication process as an essential component of a seed system for cassava and a prerequisite for rapidly disseminating improved materials is not yet established in most African countries. The common practice, dissemination of planting materials through an exchange of cuttings between farmers, is associated with the risk of, and, likely, the cause of, cassava brown streak virus disease (CBSD) and cassava mosaic virus disease (CMD) spread in Africa. The introduction and rapid distribution of Sri Lankan cassava mosaic virus (SLCMV) in South-East Asia [13], with dramatic impact on the national economies of Vietnam, Laos, Cambodia, and Thailand, and the emergence of diseases with unknown aetiologies and cures, cassava witch’s broom disease (CWBD) [14], cassava frogskin disease (CFSD) [15], and cassava root necrosis disease (CRND) [16], provide ample evidence of the constant threat of new diseases that can have severe impact on the production of cassava. Thus, there is an urgent need for a formalized seed system and propagation schemes to furnish farmers with clean planting materials at a low cost. Ideally, this would be close to farmers for easy access. The Kitovu Jani method could offer the solution due to its simplicity, quickness, and cost-effectiveness in commercial cassava seed rapid multiplication.

Cassava seed would be a secure propagule to start a healthy crop; however, for cassava propagation this is not feasible, hence its use in breeding projects with crossings objectives only [12,17]. Vegetative propagation, which involves taking three-node cuttings of cassava stem, is widely used to multiply cassava in the field [6]; however, aside from the risk of propagation of diseased material, it has a low multiplication ratio because only hardwood, lignified stems, are used as cuttings. While it takes 8–12 months to provide lignified stems for propagation, the potential to use the upper green plant parts of cassava for propagation, however, remains untapped.

Tissue culture is an efficient method of propagating cassava plants [18,19] and meristem culture [20] can be combined effectively with in vitro thermotherapy [21,22] to eliminate pathogens and to produce healthy plantlets free from pathogens. However, because in vitro culture techniques require specific infrastructure and experienced staff, it is reserved for institutions with sufficient funding and support.

For rapid propagation of cassava, yam, and other crops [23], SAH (SAH, SAHtecno, Friendswood, TX, USA) has been taken up by IITA. By use of this technique, the multiplication ratio increased dramatically from 1:5 to 1:10 (mother/offspring) to many hundreds/thousands per unit [10]. The SAH method comprises cassava’s initial in vitro multiplication followed by the rapid multiplication of single-node cuttings in SAH. These are subsequently transferred to soil for hardening under greenhouse conditions or planted directly into the field. The technique has an enormously high multiplication rate; hundreds to thousands of plantlets can be produced on a small surface within a year.

Similarly, the International Center for Tropical Agriculture (CIAT) has developed a hydroponic system to induce cassava rooting. It has advanced to propagate cassava in aeroponic cultures using a unique nutrient solution [24]. Despite the low costs per propagule, both methods require specific infrastructure and growing media that are not generally available. Furthermore, high losses of SAH cassava in the field warrant a prolonged intermediate hardening phase to produce vigorous plantlets that withstand the harsh conditions in the field.

The use of 2–3-node cuttings from nonlignified stems for propagation was reported by [25] and further modified by sprouting cassava stems in sandy soil beds and simple plastic tunnels to maintain high humidity [26,27]. The method has been adopted by several institutes in Laos, Cambodia, and Vietnam to produce high numbers of healthy cassava cuttings.

The propagation method we have developed is based on the leaf bud method developed at Empraba, Brazil [11], for cassava propagation. In contrast to the early report, we used nodal cuttings excised from green stems of cassava at 2 MAP. We concentrated on the principle and tried to simplify the process by leaving out any specific media or chemical treatments. Simply rooting single-node cuttings in water and transplanting them to soil provides a low-cost alternative for cassava multiplication accessible to all.

Our brief report underlines the feasibility of employing this method to accelerate cassava production for multiplication in breeding or virus screening projects. Additionally, it provides a comparison of propagation methods for cassava, encompassing not only the traditional multiplication through stem cuttings but also propagation via tissue culture.

Kitovu Jani can play a crucial role in the rapid production of disease-free materials. Nodal cuttings from cassava taken already two months after planting can serve as starting materials. Development of shoots and roots is within two weeks only and subsequent transfer into soils results in sturdy plantlets 6–8 weeks after the cuttings were taken. The unique feature is that only water is used for germination and no other material is needed than a few plastic containers and soil. Care must be taken that there is no microbial growth during germination, which is simply taken care of by replacing the water every two days, especially at high temperatures above 30 °C, which also shows that less favorable conditions are feasible for cassava propagation using this method. The number of plantlets that can be generated from unlignified stems of cassava depends on the plant’s age; thus, 6–8 plantlets per stem are just a rough estimate. Although Kitovu Jani has a varietal response, as we observed from both the Tanzania and Congo preliminary experiments, being cost-effective is realistic for sustainability. Taking into consideration the low survival rate in some genotypes, bearing in mind the cleanness of the water could lead to improved growth. Also, early planting before rooting could be considered, like in the TARI experiment, to overcome the late-rooting genotypes, as it had no effect on the growth of the plantlets in a later stage.

We now use Kitovu Jani as the propagation method for cassava in our research institutes and we find that the simplicity of the method is linked with robustness and feasibility of transfer to the field. More cassava genotypes need to be subjected to this propagation method and the final transfer to the open field and evaluation of the field performance of Kitovu Jani material will then allow a final assessment of the method and its economics. While the enormous multiplication ratio of SAH cannot be topped, Kitovu Jani captivates simplicity, accessibility, cost-effectiveness, and robustness which are considered prerequisite to adoption.

## 4. Materials and Methods

### 4.1. Cassava Genotypes

Various cassava genotypes were used for experiments conducted under controlled greenhouse conditions in DSMZ greenhouses and at ambient climates at African partner institutes. At DSMZ, 149 seeds (Table S1) from crosses between cassava parental lines with particular virus resistance were obtained from the cassava breeding program at IITA. The seeds underwent a hot-water treatment at 65 °C for 3 min to enhance the germination rate. Subsequently, the treated seeds were seeded into the soil and maintained in a growth chamber at 38 °C and 70% humidity. In cases where seeds failed to germinate, the hot-water treatment was repeated to increase the chance of alleviating the seeds dormancy. Once the seedlings had emerged and 3–5 leaves had developed in approximately a period of 2 weeks, the plantlets were transferred into pots (10 × 8) and moved to a foil tunnel at high humidity in the glasshouse for acclimatization. Cassava seedlings were transferred out of the foil tunnel at 4 weeks after planting and kept for 6 to 8 weeks in glasshouse under ambient conditions at 26 to 30 °C with additional light provided until approximately 10–12 fully matured leaves had developed.

At the Tanzania Agricultural Research Institute (TARI) and the International Institute of Tropical Agriculture (IITA), Tanzania, and DR Congo, the cut-and-grow technique was tried with different cassava varieties that were grown under field conditions. At IITA Tanzania, the cassava varieties Albert, Pwani, Kizmbani, Mkuranga1, Chereko, and Kiroba were used, while at TARI, cassava breeding lines including DSC 493 (12-1), DSC 525 (525), KBH2016B/504, and KBH2016B/521, were subjected to rapid propagation. At IITA Kalambo, DR Congo, Kivu Jani was tried for multiplication of the improved varieties Illona and Lumonu, recently released for resistance against root necrosis, and the variety Mudola with virus resistance. At AVPD, the breeding lines KBH2016B/521, DSC 510, DSC 525, and DSC 673 were used.

### 4.2. Cut, Grow, and Root Procedure

#### 4.2.1. Single-Node Preparation

**Figure 3:**
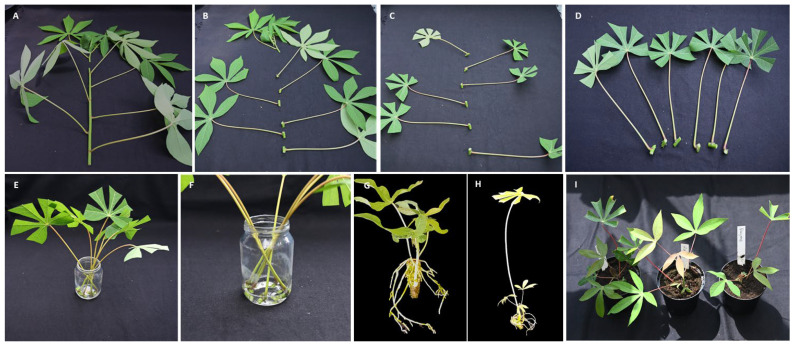
Cassava propagation of the Kitovu Jani method workflow at the DSMZ Plant Virus Department. Single nodes with a leaf attached excised from green stems without the apical part (**A**,**B**), leaves surfaces were shortened (**C**,**D**). Single nodes with leaves were placed in glass jar (**E**) with only a bit of water (**F**). Sprouts and roots germinated from a single bud node (**G**,**H**). New sprouts were transferred to soil (**I**).

For cassava propagation, nonlignified green stems from seedlings were chosen. The explants of approximately 1 cm comprised one node, petiole, and leaf, and were harvested for rooting using garden scissors (Figure 3A,B).

From a seedling with a single 50 cm stem, approximately 6 to 8 nodal cuttings were taken for each genotype (Figure 3A–D). Two nodes and leaves were kept at the rootstock to sprout and develop new branches. Apical sections with the topmost unfolded leaves were left out because their immature buds did not develop well or rotted.

#### 4.2.2. Rooting

To initiate the rooting process, the leaf surfaces of the selected cuttings were shortened to reduce evaporation (Figure 3C,D). The prepared cuttings were then placed in a glass jar or plastic container and sufficient tap water was added to ensure that nodal sections were covered (Figure 3C–F).

#### 4.2.3. Management of Containers

Consideration was given to prevent an overloading of rooting containers with cuttings. Furthermore, the water level was maintained just to cover the nodal sections (Figure 3E,F) because submersion critically delayed sprouting. Nodal cuttings were placed under greenhouse conditions and water adjustments were made when necessary. Occasional additions or changes in water were carried out, particularly when contamination from microbial growth became apparent. Special attention was paid to avoid excess water, ensuring that the water level remained in line with the nodal cuttings.

#### 4.2.4. Transplanting and Plantlet Maintenance

After the buds had sprouted and new shoots and roots had developed (approximately four weeks) (Figure 3G,H), the plantlets were transferred into pots (10 × 8) and kept under glasshouse conditions (Figure 3I).

### 4.3. Cut, Grow, and Root under African Conditions

All partners at TARI, IITA, and AVPD followed similar procedures. Nodal explants essentially prepared as described above (Figure 3A–D) were placed in plastic jars or similar containers and left for germination until leaves and roots had developed. Thereafter, the plantlets were transferred into plastic growth bags (2L) filled with soil. All propagation was conducted under ambient conditions of a screenhouse while at IITA, Kalambo, a SAH laboratory was used to keep the cultures under artificial light.

The diverse environments, cassava genotypes, and growing conditions provided a good indication of the overall feasibility of this method for rapid multiplication of cassava.

## 5. Conclusions

Rapid replacement of cassava planting materials with improved materials is imperative to ensure that farmers profit quickly from breeding advancements and, furthermore, to achieve and maintain an acceptable phytosanitary status of the crop to provide healthy and vigorously growing planting materials. Efficient methods for cassava multiplication are essential to provide farmers with cost-effective planting materials at scale. Accessibility is key for acceptance and adoption, especially in African conditions with low infrastructural, financial, and personal obstacles. The Kitovu Jani technique, which we present here, has proven successful for rapidly producing clones from individual cassava seedlings, offering a promising solution to advance speed breeding of cassava genotypes for virus resistance and other traits by drastically reducing the length of the breeding cycle.

Our demonstration revealed that propagating cassava using single-node cuttings from seedlings, grown under glasshouse conditions, achieved almost 100% success from germination to plantlets in soil. Field conditions in Africa showed varying success, with notable performance from varieties like Pwani, TARICASS4, and Mkuranga1. While further optimization and adaptation to local conditions are possible, the method outperformed currently used multiplication methods in terms of simplicity, speed, and cost-effectiveness. Although it may not match the multiplication ratios of some methods, its adaptability to various genotypes and feasibility for field transfer make it a compelling choice.

In conclusion, the Kitovu Jani technique is a promising avenue for advancing cassava breeding, providing a practical solution for accelerating the production of healthy planting materials. While acknowledging the need for further optimization and adaptation to local conditions, this method holds immense potential for transforming cassava cultivation, promoting enhanced crop health, and, ultimately, benefiting farmers and communities.

## Data Availability

Data are contained within the article and Supplementary Materials.

## Supplementary Materials

The following supporting information can be downloaded at: https://www.mdpi.com/article/10.3390/plants13040471/s1, Table S1: Cassava seedlings used in the propagation at DSMZ Plant Virus Department.
