# Supplementary material for: Cut, Root, and Grow: Simplifying Cassava Propagation to Scale
Source: Plants (Basel). 2024 Feb 6;13(4):471. doi: 10.3390/plants13040471 (PMC10893366; doi:10.3390/plants13040471)
Supplement: Supplementary file 1 [file plants-13-00471-s001.zip › plants-2689005-supplementary.pdf]

plants

Table S1. Cassava seedlings used in the propogation at DSMZ-plant virus department.

Population 4	nr.	Pedigree	Accession_Name
	1	COL 40 x KBH2016B/504	POP401
	2		POP402
	3		POP403
	4		POP404
	5		POP405
	6		POP406
	7		POP407
	8		POP408
	9		POP409
	10		POP4010
	11		POP4011
	12		POP4012
	13		POP4013
	14		POP4014
	15		POP4015
	16		POP4016
	17		POP4017
	18		POP4018
	19		POP4019
	20		POP4020
	21		POP4021
	22		POP4022
	23		POP4023
	24		POP4024
	25		POP4025
	26		POP4026
	27		POP4027
	28		POP4028
	29		POP4029
	30		POP4030
	31		POP4031
	32		POP4032
	33		POP4033
	34		POP4034
	35		POP4035
	36		POP4036
	37		POP4037
	38		POP4038
	39		POP4039
	40		POP4040
	41		POP4041
	42		POP4042
	43		POP4043
	44		POP4044
	45		POP4045
	46		POP4046

47		POP4047
48	COL40 x TMEB-14	POP4048
49		POP4049
50		POP4050
51		POP4051
52		POP4052
53		POP4053
54		POP4054
55		POP4055
56		POP4056
57		POP4057
58		POP4058
59		POP4059
60		POP4060
61		POP4061
62		POP4062
63		POP4063
64		POP4064
65		POP4065
66		POP4066
67		POP4067
68		POP4068
69		POP4069
70		POP4070
71		POP4071
72		POP4072
73		POP4073
74		POP4074
75		POP4075
76		POP4076
77		POP4077
78		POP4078
79		POP4079
80		POP4080
81		POP4081
82		POP4082
83		POP4083
84	COl40 x MM16/1487	POP4084
85		POP4085
86		POP4086
87		POP4087
88		POP4088
89		POP4089
90		POP4090
91		POP4091
92		POP4092
93		POP4093
94		POP4094
95		POP4095
96		POP4096

97	POP4097
98	POP4098
99	POP4099
100	POP40100
101	POP40101
102	POP40102
103	POP40103
104	POP40104
105	POP40105
106	POP40106
107	POP40107
108	POP40108
109	POP40109
110	POP40110
111	POP40111
112	POP40112
113	POP40113
114	POP40114
115	POP40115
116	POP40116
117	POP40117
118	POP40118
119	POP40119
120	POP40120
121	POP40121
122	POP40122
123	POP40123
124	POP40124
125	POP40125
126	POP40126
127	POP40127
128	POP40128
129	POP40129
130	POP40130
131	POP40131
132	POP40132
133	POP40133
134	POP40134
135	POP20135
136	POP20136
137	POP20137
138	POP20138
139	POP20139
140	POP20140
141	POP20141
142	POP20142
143	POP20143
144	POP20144
145	POP20145
146	POP20146

147	POP20147
148	POP20148
149	POP20149
